# Clinical Predictors of Response to Cognitive-Behavioral Therapy in Pediatric Anxiety Disorders: The Genes for Treatment (GxT) Study

**DOI:** 10.1016/j.jaac.2015.03.018

**Published:** 2015-06

**Authors:** Jennifer L. Hudson, Robert Keers, Susanna Roberts, Jonathan R.I. Coleman, Gerome Breen, Kristian Arendt, Susan Bögels, Peter Cooper, Cathy Creswell, Catharina Hartman, Einar R. Heiervang, Katrin Hötzel, Tina In-Albon, Kristen Lavallee, Heidi J. Lyneham, Carla E. Marin, Anna McKinnon, Richard Meiser-Stedman, Talia Morris, Maaike Nauta, Ronald M. Rapee, Silvia Schneider, Sophie C. Schneider, Wendy K. Silverman, Mikael Thastum, Kerstin Thirlwall, Polly Waite, Gro Janne Wergeland, Kathryn J. Lester, Thalia C. Eley

**Affiliations:** a Centre for Emotional Health, Macquarie University, Sydney, Australia; b King’s College London, Institute of Psychiatry, Psychology and Neuroscience, Medical Research Council (MRC) Social, Genetic and Developmental Psychiatry (SGDP) Centre, London; c University of Aarhus, Denmark; d Research Institute Child Development and Education, University of Amsterdam; e School of Psychology and Clinical Language Sciences, University of Reading, UK; f University Medical Center Groningen, University of Groningen, The Netherlands; g Institute of Clinical Medicine, University of Oslo, Norway and Anxiety Research Network, Haukeland University Hospital, Bergen, Norway; h Ruhr-Universität Bochum, Germany; i University Koblenz-Landau, Landau, Germany; j University of Basel, Switzerland; k Yale University, New Haven, CT; l MRC Cognition and Brain Sciences Unit, Cambridge, UK; m Ruhr-Universität Bochum, Germany; n Anxiety Research Network, Haukeland University Hospital; o School of Psychology, University of Sussex, UK

**Keywords:** anxiety disorders, treatment, predictors, cognitive-behavioral therapy

## Abstract

**Objective:**

The Genes for Treatment study is an international, multisite collaboration exploring the role of genetic, demographic, and clinical predictors in response to cognitive-behavioral therapy (CBT) in pediatric anxiety disorders. The current article, the first from the study, examined demographic and clinical predictors of response to CBT. We hypothesized that the child’s gender, type of anxiety disorder, initial severity and comorbidity, and parents’ psychopathology would significantly predict outcome.

**Method:**

A sample of 1,519 children 5 to 18 years of age with a primary anxiety diagnosis received CBT across 11 sites. Outcome was defined as response (change in diagnostic severity) and remission (absence of the primary diagnosis) at each time point (posttreatment, 3-, 6-, and/or 12-month follow-up) and analyzed using linear and logistic mixed models. Separate analyses were conducted using data from posttreatment and follow-up assessments to explore the relative importance of predictors at these time points.

**Results:**

Individuals with social anxiety disorder (SoAD) had significantly poorer outcomes (poorer response and lower rates of remission) than those with generalized anxiety disorder (GAD). Although individuals with specific phobia (SP) also had poorer outcomes than those with GAD at posttreatment, these differences were not maintained at follow-up. Both comorbid mood and externalizing disorders significantly predicted poorer outcomes at posttreatment and follow-up, whereas self-reported parental psychopathology had little effect on posttreatment outcomes but significantly predicted response (although not remission) at follow-up.

**Conclusion:**

SoAD, nonanxiety comorbidity, and parental psychopathology were associated with poorer outcomes after CBT. The results highlight the need for enhanced treatments for children at risk for poorer outcomes.

This article presents the phenotypic analyses from the Genes for Treatment (GxT) study, an international multisite collaboration exploring the role of genetic and clinical predictors of response to cognitive-behavioral therapy (CBT) in pediatric anxiety disorders. Here we present analyses examining clinical predictors of outcome. Our research focuses on anxiety disorders, as these are the most prevalent mental disorders, and, when experienced early, are associated with increased risk of multiple disorders later in life.^1^ Although CBT has been established as an efficacious treatment, roughly 40% of children retain their disorder after treatment.^2,3^ Identifying predictors of outcome, including both response (change in symptoms) and rates of remission, may allow clinicians to identify children at risk for poorer outcomes before they commence therapy^4^ and help guide the development of more effective treatments for these children.

There is some evidence to suggest that a diagnosis of social anxiety disorder (SoAD), and comorbid mood and externalizing disorder/symptoms are each associated with poorer treatment outcomes.^5-8^ Parental depression and anxiety have also been associated with poorer response and remission. Nevertheless, findings for each of these predictors are inconsistent. Indeed, the 2 most recent systematic reviews of the literature failed to find conclusive evidence for a role of any of these factors in treatment outcome.^9,10^

It is likely that the absence of multivariate models, in conjunction with small samples, varied methodology, and the failure to consistently distinguish between response and remission, has contributed to the inconsistent results. The collaboration of multiple sites and trials can overcome these limitations. This study represents the largest collaboration to date of pediatric anxiety treatment data and provides significantly greater power to detect genetic, clinical, and demographic predictors of outcome than previously possible. The sample includes data from previously published studies,^11,12^ as well as data from ongoing trials yet to be published. The goal of the current article was to identify clinical and demographic predictors of outcome. We present results from a linear mixture model with a higher-order random effect allowing individuals to be nested within trials, thereby controlling for possible trial and site differences. The design allows for the simultaneous examination of multiple variables, resulting in the identification of unique predictors. In our prior analyses on a subset of the current sample, we found that being female, greater initial anxiety severity, and the presence of comorbid mood and externalizing disorders were uniquely associated with poorer response to CBT.^13^ Although these findings are largely consistent with the literature, the finding that girls do worse than boys in CBT for child anxiety has emerged in only 1 individual trial^14^ and requires further examination in a larger sample. We hypothesized that female gender, the presence of SoAD, comorbid mood disorder, or externalizing disorders, and greater parental psychopathology would predict poorer outcomes to CBT in pediatric anxiety.

## Method

### Sample

The Genes for Treatment study (GxT) sample comprises data from 1,519 children who received a course of CBT for anxiety at 1 of 11 sites: Sydney, Australia (n = 706); Reading, UK (n = 340); Aarhus, Denmark (n = 124); Bergen, Norway (n = 119); Bochum, Germany (n = 57); Basel, Switzerland (n = 49); Groningen, the Netherlands (n = 37); Oxford, UK (n = 21); Miami, Florida, USA (n = 50); Cambridge, UK (n = 12) and Amsterdam, the Netherlands (n = 4). Participants were included if they were 5 to 18 years of age (94% were 5–13 years of age), met *DSM-IV* criteria for a primary diagnosis of an anxiety disorder, and provided a DNA sample. Parents gave written consent, and children gave written or verbal assent. Exclusion criteria were significant physical or intellectual impairment or psychosis.

All participants received individual-based CBT involving a single child (with or without their parent; n = 426, 28%; mean [SD] number sessions = 11.8 [3.2]), group-based CBT (n = 800, 52.7%; mean [SD] number sessions = 10 [0]) or guided CBT self-help (n = 293, 19.3%; mean [SD] number sessions = 7.3 [1.5]) and provided at least 1 posttreatment assessment.

All treatments were manualized, and treatment protocols across all sites were comparable for core elements of CBT including teaching of coping skills, cognitive restructuring, and exposure. Further details are provided in Supplement 1, available online.

### Measures

All sites administered the Anxiety Disorders Interview Schedule for *DSM-IV*, Parent and Child Versions (ADIS-IV-C/P^15^) except at Bochum and Basel, where the Diagnostisches Interview bei psychischen Strungen im Kindes- und Jugendalter (Kinder-DIPS) was used.^16^ Participants were assessed before and immediately after treatment (posttreatment), with further assessments made 3, 6, or 12 months after treatment cessation where possible (follow-up). The presence and severity of the primary anxiety disorder was measured at each time point. Severity was assessed using the clinician severity rating (CSR) from the structured interview, which assigns a score of 0 to 8 (absent to very severe). A diagnosis was made when the child met the diagnostic criteria and received a CSR of 4 or more, usually based on a composite of parent and child report. Diagnoses were made according to *DSM* criteria.^17^

Ten sites (Sydney, Reading, Aarhus, Bochum, Basel, Groningen, Oxford, Florida, Cambridge, and Amsterdam) also assessed comorbid mood (major depression or dysthymia) or externalizing disorders (oppositional defiant disorder, conduct disorder or attention-deficit/hyperactivity disorder [ADHD]) at baseline using the ADIS-C/P.

In addition, at 8 sites (Sydney, Reading, Aarhus, Bergen, Bochum, Oxford, Florida, Amsterdam), parents completed the Depression Anxiety Stress Scales (DASS),^18^ assessing depression, anxiety, and stress symptoms experienced over the past week. For this study, the 3 subscales were summed to create an overall measure of parental psychopathology.

All assessments were completed by graduate assistants or clinical staff (mainly psychologists) trained in the administration of the instruments. Sites have previously reported good interrater reliability for the diagnostic instruments using these samples.^11,19,20^

### Statistical Analysis

To make use of all available postbaseline assessments and to provide estimates in the presence of missing values, the effects of predictors on outcome were tested using mixed models fitted with full maximum likelihood. All models included the fixed effects of baseline severity (CSR score of the primary diagnosis at baseline, centered at the mean) and the linear and quadratic effects of time to account for the curvilinear slope of treatment outcome. To account for correlations between repeated measures from the same participant, all models included the random effects of individual. We also included a higher-order random effect of trial to account for between-trial differences in outcome. As each trial was conducted at a single site, this random effect also accounted for between-site differences. Predictor variables were entered simultaneously. Thus, when a significant predictor is identified, it is significant over and above the other predictors in the model.

We conducted analyses using 2 treatment outcomes: response (change in diagnostic severity), and remission (absence of the primary diagnosis). In response analyses, linear mixed-effects models were used to investigate the effects of baseline predictor variables on change in severity (CSR score) of the primary anxiety diagnosis from baseline at each time point. In these analyses, the β values of variables predicting a more favorable response to treatment (i.e., greater reduction in severity) are negative, whereas variables predicting a less favorable response are positive.

In analyses of remission, similar logistic mixed effects models were used to investigate the effects of baseline predictor variables on absence of the primary anxiety diagnosis at each time point. In these analyses, odds ratios of variables predicting a higher likelihood of remission (a loss of the primary diagnosis) are greater than 1, whereas variables predicting a lower likelihood of remission have odds ratios of less than 1.

Initially, we considered response and remission using data from the entire duration of the trial. However, the power of the GxT sample also enabled us to compare the results from separate analyses predicting outcome at the posttreatment and follow-up assessments, respectively. To formally test whether predictors were specific to the posttreatment or follow-up time points, we also tested the significance of a time-by-predictor interaction in models using data from each time point in the study.

Our primary analyses included only the clinical and demographic data collected in all of the participating sites as predictors of treatment outcome. These were treatment type (in which group-based and guided self-help CBT were each compared with individual-based CBT), age (centered at the mean), gender (0 = male, 1 = female), and primary diagnosis (in which SoAD, separation anxiety disorder [SAD], specific phobia [SP], and “other anxiety” disorders were each compared with generalized anxiety disorder [GAD]). In secondary analyses, we also explored the effects of comorbid mood and externalizing disorders, and parental psychopathology as predictors of outcome in trials where these data were available. These analyses included the presence of a comorbid mood or externalizing disorder and standardized total score of the DASS to indicate parental depression, anxiety, and stress symptoms. To control for multiple testing (6 analyses), we considered predictors significant at a Bonferroni-corrected α level of 0.008 (0.05/6).

Finally, our approach to measuring outcome allowed us to include all of the available data and provided increased power to detect predictors of treatment outcome. To ensure that our findings were comparable to previous analyses of treatment outcome, we also conducted analyses exploring the effects of the factors included above on change in severity scores and absence of primary anxiety disorder for each time point separately, using linear and logistic regression, respectively.

All statistical analyses were conducted using STATA version 12.0.^21^

## Results

### Sample Characteristics

**Figure 1:**
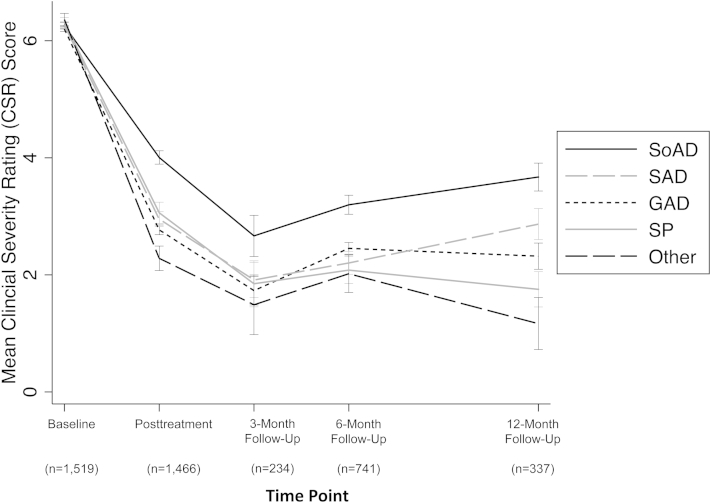
Mean clinician severity rating (CSR) score by primary diagnosis at each time point. Note: Error bars represent 1 standard error of the mean; “other” includes panic disorder with and without agoraphobia and agoraphobia without panic disorder (n = 38), obsessive-compulsive disorder (OCD; n = 44), posttraumatic stress disorder (PTSD; n = 17), selective mutism (n = 4), or anxiety disorder not otherwise specified (n = 10). GAD = generalized anxiety disorder; SAD = separation anxiety disorder; SoAD = social anxiety disorder; SP = specific phobia.

The baseline demographic and clinical characteristics of the sample overall and by site are given in Table 1. The most common primary diagnosis was GAD, followed by SoAD, SAD, and SP. The remaining participants met criteria for “other anxiety” disorders, which included panic disorder with and without agoraphobia and agoraphobia without panic disorder (n = 38), obsessive-compulsive disorder (OCD; n = 44), posttraumatic stress disorder (PTSD; n = 17), selective mutism (in patients with primary selective mutism, a diagnosis of severe SoAD was also given; the selective mutism was considered by the clinician to be primary, the most interfering: n = 4) or anxiety disorder not otherwise specified (n = 10). CSR scores for the primary diagnosis indicated that the majority of the sample (79.1%) were rated 6 or above (either severe or very severe) at the start of treatment. Changes in severity scores grouped by primary diagnosis with the n for each time point are given in Figure 1. The 10 sites that measured comorbid mood and externalizing disorders found both to be common, with prevalence rates of 10.7% and 18.2%, respectively. Parental psychopathology was also common, with 114 (8.9%), 137 (10.6%), and 172 (13.3%) scoring above the cut-offs for severe depression (21+), anxiety (15+), and stress (26+), respectively.^22^ Individuals treated with group CBT and those treated with self-help CBT had significantly more missing outcome data than those treated with individual CBT (β = 0.26, 95% CI = 0.14–0.37, *p* < .001 and β = 0.42, 95% CI = 0.29–0.55, *p* < .001, respectively). However, missingness was not associated with baseline severity, age, gender, primary diagnosis, comorbid mood or externalizing disorders, or parental psychopathology (all *p* values >.1).

### Predictors of Response and Remission

Results of the linear and logistic mixed models used to explore the effects of demographic and clinical characteristics on response (change in diagnostic severity of the primary diagnosis from baseline) and remission (absence of the primary diagnosis) are given in Tables 2 and 3. Outcome was considered first using data from all time points, then at the posttreatment or follow-up assessments specifically. To assess response (change in diagnostic severity), baseline severity of diagnosis was included in all models as a covariate. Higher baseline severity was associated with higher severity scores and a lower likelihood of remission across all time points.

### Treatment Type

Treatment type was not associated with response or remission overall or in analyses conducted using the posttreatment or follow-up assessments separately.

### Gender and Age

Gender and age did not significantly predict response or remission after correction for multiple testing. Age-by-gender interactions were also nonsignificant.

### Primary Diagnosis

Primary diagnosis was significantly associated with both response and remission. In response analyses, individuals with SoAD showed significantly less change in their diagnostic severity than those with GAD. Correspondingly, in analyses of remission, individuals with primary SoAD were significantly more likely to retain their diagnosis at posttreatment and follow-up assessments than those with GAD. Findings were similar regardless of time point. This suggests that this factor had similar effects on outcome at both the posttreatment and follow-up time points. The effects of SoAD were also similar regardless of treatment type. Results of response analyses for individual CBT, group CBT, and guided self-help respectively were as follows: β = 0.36, 95% CI = 0.16–0.56, *p* = .001; β = 0.54, 95% CI = 0.37–0.70, *p* < .001; and β = 0.37, 95% CI = 0.13–0.62, *p* = .003. Results of remission analyses for individual CBT, group CBT, and guided self-help respectively were as follows: OR = 0.41, 95% CI = 0.18–0.93, *p* = .033; OR = 0.16, 95% CI = 0.07–0.33, *p* < .001; OR = 0.08, 95% CI = 0.01–0.84, *p* = .035. This suggests that the poor outcomes for individuals with SoAD were not driven by a poor response to a particular treatment type. We also found that the presence of SoAD anywhere in the child’s profile significantly worsened his/her outcome (see Table S1, available online).

Individuals with a primary diagnosis of SP showed significantly poorer response (less change in severity) and lower rates of remission than those with GAD. However, these findings were specific to outcome at the posttreatment assessment. To test the statistical significance of these effects, we included a diagnosis by study period interaction in analyses using data from all time points. This interaction was significant for both response and remission outcomes (β = –0.17, 95% CI = –0.31 to 0.02, *p* = .025 and OR = 2.33, 95% CI = 1.15–4.71, *p* = .035, respectively), indicating that SP becomes a less important predictor of outcome in the later stages of the study.

### Comorbid Mood or Externalizing Disorders

Using the same models and covariates as above (that is, baseline severity as a covariate and gender, age, primary diagnosis, and treatment type as predictors), we conducted secondary analyses, exploring the effects of comorbid mood or externalizing disorders, and parental psychopathology on remission and response in trials that measured these factors (lower portion of Tables 2 and 3). Effect sizes for treatment type, age, gender, and diagnosis were similar to those in the previous models (see Table S2, available online, for estimates from the full model).

The presence of a comorbid externalizing disorder was associated with a poorer response to treatment (less change in severity). Although these effects appeared to be smaller in the follow-up than in the posttreatment assessments, externalizing disorder by study period interactions were nonsignificant (all *p* values >.1), indicating that this factor had similar effects at both outcome time points. Although findings were nominally significant (*p* < .05) for remission, they did not withstand correction for multiple testing.

The presence of a comorbid mood disorder was also associated with poorer response (especially at posttreatment) and lower likelihood of remission using data from all time points. However, as with externalizing disorders, a mood disorder by study period interaction was nonsignificant for response and remission (all *p* values >.1), suggesting that the effects of mood disorders did not differ across time.

### Parental Psychopathology

Parental psychopathology (total DASS score) was associated with significantly poorer response, particularly in the follow-up assessment. There was a significant parental psychopathology by study period interaction (β = 0.11, 95% CI = 0.06–0.15, *p* < .001), indicating that these effects were specific to follow-up. Although findings were in a similar direction for remission, these results did not withstand correction for multiple testing.

### Parental Involvement and Treatment Length

A recent individual-level meta-analysis combining data from published child anxiety treatment trials suggested that the level of parental involvement in treatments may have an impact on outcome. Specifically, treatments that involved parents and used contingency management strategies and/or a transfer of control model showed better outcomes than treatments that included other types of parental involvement.^23^ To account for these effects in the current study, we coded parental involvement in each trial using the same approach as in this previous study (i.e., low involvement, active involvement without emphasis on contingency management, and transfer of control or active involvement with emphasis on contingency management or transfer of control) and reanalyzed the data using parental involvement as a covariate (see Supplement 1 Methods and Table S3, available online). The results indicated that the level of parental involvement was not associated with either response or remission, and the inclusion of this variable did not affect our previous findings. As the included trials differed in the number of planned sessions, we also explored whether treatment length explained our previous findings (see Table S4, available online). These analyses suggested that treatment length was not associated with either response or remission and did not confound the relationship between diagnosis, comorbidity, and parental psychopathology and outcome.

### Comparison With Previous Analyses

We previously reported on clinical and demographic predictors of treatment outcome at follow-up for a subset (n = 384) of this sample. These analyses suggested that female gender, greater initial anxiety severity, and comorbid mood and externalizing disorders were all associated with poorer response to CBT.^13^ To ensure that the findings in the current report were not driven entirely by data from these previous analyses, we reanalyzed the GxT data excluding this subset. The results were similar to those from the entire sample (see Table S5, available online).

We chose to focus on outcomes for the primary anxiety disorder in the current article, as this is typically the main outcome measure used in clinical trials and is typically the target of treatment. Nevertheless, some previous studies have considered remission as an absence of all anxiety diagnoses. When we conducted analyses using this stricter definition, the results were comparable to our previous findings of remission from the primary diagnosis (see Table S6, available online). That is, individuals with a diagnosis of SoAD were significantly less likely to experience remission. The presence of comorbid externalizing disorders was also significantly associated with lower likelihood of remission, and although findings were only nominally significant (*p* < .05) for mood disorders and parental psychopathology, they followed the same pattern as we observed in our original analysis.

Our longitudinal approach to measuring outcome allowed us to include all of the available data, providing increased power to detect predictors of outcome. To enable direct comparisons with other studies, we also conducted analyses of remission of the primary diagnosis and change in the severity of primary diagnosis at each time point in the study (see Tables S7 and S8, available online). Although findings did not reach statistical significance at all time points because of the lower power inherent to subgroup analyses, the results are similar to those from the mixture model approach.

### Sensitivity Analyses

We combined data from multiple trials including patients with a wide age range with a variety of different primary disorders. This approach provided us with increased power to detect small effects and to identify robust predictors with the highest potential clinical utility. Nevertheless, the resulting heterogeneity may have affected the results. To address this we conducted a series of sensitivity analyses in which we attempted to reduce the heterogeneity of the sample to include only individuals within a narrower age range (5–13, n = 1,429, 94.1%: see Table S9, available online), only those with the 4 most common diagnoses (GAD, SoAD, SAD, and SP; n = 1,406, 92.5%; see Table S10, available online), or only those who received a treatment that was not diagnosis specific (n = 1,423, 93.7%; see Table S11, available online). In each of these analyses, findings were equivalent to those from the full analysis, suggesting that they were not the result of excessive heterogeneity in the sample.

## Discussion

This is the first article to emerge from an international multisite collaboration exploring the role of genetic and clinical predictors of outcome after CBT for pediatric anxiety disorders. We identified several clinical predictors of outcome, some of which showed effects only at specific time points. As our study examined multiple treatment predictors, we were able to identify variables that contributed unique variance over and above other predictors, such as baseline severity.

In support of previous research,^5,6^ children with primary SoAD had the poorest outcomes and were nearly twice as likely as children with primary GAD to still have a diagnosis at the end of the study. This is not to say that CBT is ineffective for children with SoAD but, rather, it suggests that CBT is less effective than for children with other types of anxiety disorders. These results cannot be explained by initial severity, comorbidity, or parental psychopathology. Currently we do not fully understand why children with SoAD have poorer outcomes. Future research must endeavor to understand the mechanisms underlying these poorer outcomes for children with SoAD and to develop and evaluate enhanced treatments. Of particular note, the treatment delivered in the trials reported here were typically of a generic format targeting a heterogeneous group of children with anxiety disorders (with the exception of patients with PTSD from the Cambridge site and patients with SAD from the Bochum and Basel sites). Treatments tailored specifically to target SoAD (e.g., increased social skills training, video feedback, attention training) such as Social Effectiveness Training^24^ may enhance outcomes for this more intractable disorder. Unexpectedly, children with a primary diagnosis of SP also showed poorer outcomes immediately after treatment than children with a primary diagnosis of GAD, but this difference disappeared by follow-up. This suggests that children with a primary diagnosis of SP take longer to demonstrate equivalent outcomes to children with GAD. It is possible that, given the graded approach to exposure, severe fears were tackled toward the end of therapy after other, less severe comorbid fears were reduced.

Children presenting with comorbid mood or externalizing disorders were approximately twice as likely as those without to retain their primary anxiety disorder across all outcome time points. These children also demonstrated reduced levels of symptom change. These results support the growing body of evidence that suggests that comorbidity affects outcomes of CBT for anxious youth.^7,8^ There was weak support for a temporal effect of comorbidity on outcome, with the importance of comorbid disorders reduced by the follow-up time point. Although this could be explained by reduced power within these secondary analyses, it could also suggest that children with comorbid disorders take longer to improve compared to those without, suggesting that treatments could be developed that bring about more efficient change.

Finally, these data suggest that parental psychopathology may have an impact on outcomes, specifically during the follow-up period, although this varies depending on the measure of outcome used. Parental psychopathology did not predict remission of child anxiety disorder. In contrast, when outcome was measured as change in diagnostic severity, children of parents with elevated symptoms of anxiety, depression, and stress showed poorer response at follow-up. Previous research has shown, albeit inconsistently, that increased parental psychopathology reduces the efficacy of treatment for anxious youth.^9,25^ However, the temporal effects observed here are novel and may explain why not all studies report a significant association. Moreover, these findings indicate that parental psychopathology may exert increasing influence on the child’s symptom presentation even after a child’s treatment has been completed. At the completion of treatment, children no longer rely on the therapist to monitor the successful execution of exposure tasks but, rather, become increasingly reliant on parents or themselves. Given this, it is possible that parental psychopathology could exert greater interference on the child’s symptom reduction during this period.^26^ The majority of evidence shows comparable outcomes for treatments with limited versus increased parental involvement. However, a recent individual patient data meta-analysis showed enhanced long-term outcomes for treatments with greater parent involvement and increased focus on parenting factors such as contingency management.^23^ Furthermore, there is some evidence to suggest that, in the long term, providing additional parent anxiety management may lead to enhanced outcomes for children with anxiety.^27^

Treatment modality was not a significant predictor of outcome, suggesting that treatment change is influenced by factors common to the programs included in this study. Consistent with a recent meta-analysis,^28^ age also was not a significant predictor of outcome, suggesting that CBT works just as well for younger and older children, although the majority of our sample was less than 13 years of age; thus, our conclusions with respect to adolescence are limited. Our lack of evidence for a unique effect of gender is in contrast to a previous analysis of a small subset of these data.^13^

Of note, the significance of the predictors identified cannot be explained by the child’s baseline severity of the primary diagnosis, as this was included in the models. As all variables were modeled simultaneously, predictor effects are significant over and above all other predictors in the model. Thus we can conclude, for instance, that SoAD is a significant predictor of poorer response and remission, over and above comorbid mood disorders.

This study represents the largest of its kind and was made possible through sharing of data and procedures. Despite these strengths, there are a number of limitations. First, there is considerable heterogeneity in the sample. We included trial as a covariate in our analyses and used broadly common assessment tools; yet each trial and site in which it was conducted had subtle differences in recruitment, assessment, and treatment. We also had very few adolescents in our sample, limiting generalization of our findings to preadolescent children. Future research needs to determine whether these predictors are also important for adolescents receiving treatment for anxiety disorders. As we were interested in predictors of poor outcome in children who received a full course of CBT, a control group was not appropriate. Yet it is possible that the predictors identified in this article may also be predictive of poorer outcomes even in the absence of treatment.

In summary, these findings suggest that CBT is effective for children irrespective of whether treatment is delivered in individual or group format and irrespective of the child’s age and gender. CBT is more effective for children without primary SoAD or comorbid mood disorders. Children with primary specific phobia or comorbid externalizing disorders may show slower response; however, in the long term, there should be no significant difference in outcome. Finally, the impact of having a parent with elevated levels of psychopathology will have an increasingly important impact on the child’s outcomes after treatment is complete. The next step is to develop and to evaluate enhanced treatments for children at risk for poorer outcome.

**Clinical Guidance**

• CBT works irrespective of a child’s age and gender.
• Children with SoAD had poorer outcomes compared to children with other anxiety disorders.
• Parent involvement did not impact treatment outcome.
• There is some evidence that the presence of comorbid mood disorders and parental psychopathology may lead to poorer outcomes

## Figures and Tables

**Table 1:** Baseline Characteristics of Included Participants From Each Site

Characteristic	Sydney		Reading		Aarhus		Bergen		Bochum		Basel		Groningen		Oxford		Florida		Cambridge		Amsterdam		Total	
Severity of primary diagnosis: mean (SD)	6.4	(0.9)	5.6	(0.8)	6.5	(1.2)	6.9	(1.1)	6.8	(1.1)	6	(0.8)	6.2	(1)	5.5	(1)	6.8	(1.2)	6.3	(1.2)	5.8	(1.7)	6.3	(1)
Gender: female n (%)	342	(48.4)	188	(55.3)	70	(56.5)	64	(53.8)	33	(57.9)	27	(55.1)	17	(45.9)	13	(61.9)	21	(42.9)	8	(66.7)	0	(0)	783	(51.6)
Age: mean (SD)	9.4	(1.9)	9.7	(1.9)	11	(2.4)	11.4	(2.1)	11.3	(2.5)	8.6	(2.2)	11.9	(3.1)	9	(1.8)	9.5	(2.2)	12.6	(2.8)	12	(1.8)	9.9	(2.2)
Primary diagnosis: n (%)																								
GAD	373	(52.8)	103	(30.3)	31	(25)	22	(18.5)	6	(10.5)	0	(0)	7	(18.9)	3	(14.3)	13	(26)	0	(0)	0	(0)	558	(36.7)
SoAD	151	(21.4)	67	(19.7)	18	(14.5)	54	(45.4)	15	(26.3)	0	(0)	15	(40.5)	7	(33.3)	13	(26)	0	(0)	1	(25)	341	(22.4)
SP	54	(7.6)	59	(17.4)	19	(15.3)	0	(0)	20	(35.1)	0	(0)	6	(16.2)	1	(4.8)	6	(12)	0	(0)	1	(25)	166	(10.9)
SAD	83	(11.8)	87	(25.6)	37	(29.8)	43	(36.1)	13	(22.8)	49	(100)	6	(16.2)	9	(42.9)	12	(24)	0	(0)	2	(50)	341	(22.4)
Other	45	(6.4)	24	(7.1)	19	(15.3)	0	(0)	3	(5.3)	0	(0)	3	(8.1)	1	(4.8)	6	(12)	12	(100)	0	(0)	113	(7.4)
CBT treatment: n (%)																								
Individual-based	20	(2.8)	140	(41.2)	2	(1.6)	58	(48.7)	57	(100)	49	(100)	37	(100)	0	(0)	50	(100)	12	(100)	1	(25)	426	(28)
Group-based	614	(87)	0	(0)	122	(98.4)	61	(51.3)	0	(0)	0	(0)	0	(0)	0	(0)	0	(0)	0	(0)	3	(75)	800	(52.7)
Guided self-help	72	(10.2)	200	(58.8)	0	(0)	0	(0)	0	(0)	0	(0)	0	(0)	21	(100)	0	(0)	0	(0)	0	(0)	293	(19.3)
Comorbidity: n (%)																								
Mood disorder	76	(10.8)	38	(11.3)	16	(12.9)	—a		11	(19.3)	1	(2)	1	(2.7)	1	(4.8)	2	(4)	4	(36.4)	0	(0)	150	(10.7)
Externalizing disorder	121	(17.1)	92	(27.3)	10	(8.1)	—a		3	(5.3)	2	(4.1)	2	(5.4)	4	(19)	13	(26)	6	(54.5)	1	(25)	254	(18.2)
Parental psychopathology: mean (SD)																								
Total	33.6	(18.2)	34.6	(23.2)	18.2	(14.6)	13.5	(14.6)	28.2	(16.9)	—a		—a		33	(16.2)	28.4	(12.1)	—a		22.2	(20.1)	30.8	(19.9)
Depression	7.3	(6.2)	7.8	(7.8)	2.5	(3.3)	2.3	(4.2)	4.8	(5.3)	—a		—a		7.6	(6.2)	5.2	(4.9)	—a		5.5	(6.4)	6.5	(6.5)
Anxiety	9.8	(7.7)	10.5	(9.2)	6.2	(6.4)	4.3	(7.1)	7.7	(7.8)	—a		—a		9.4	(6.7)	7.4	(4.1)	—a		3.8	(3.5)	9.1	(8)
Stress	16.5	(7.8)	16.4	(9.1)	9.5	(6.8)	6.9	(5.5)	15.8	(7.7)	—a		—a		15.9	(6.7)	15.8	(7.1)	—a		13	(11.3)	15.2	(8.4)

Note: “Other” includes panic disorder with and without agoraphobia and agoraphobia without panic disorder (n = 38), obsessive-compulsive disorder (OCD; n = 44), posttraumatic stress disorder (PTSD; n = 17), selective mutism (n = 4), or anxiety disorder not otherwise specified (n = 10). CBT = cognitive-behavioral therapy; GAD = generalized anxiety disorder; SAD = separation anxiety disorder; SoAD = social anxiety disorder; SP = specific phobia.

a Data not available for this site.

**Table 2:** Results of Linear Mixed Models Examining Predictors of Treatment Response (Change in Severity of the Primary Diagnosis From Baseline) Using Data From All Time Points, or Separately Using Only the Posttreatment or at Follow-Up Assessments

	All Time Points^a^		Posttreatment Assessment		Follow-Up Assessments^a^	
	β	(95% CI)	β	(95% CI)	β	(95% CI)
Severity of primary diagnosis at baseline	0.18	(0.14–0.21)∗	0.20	(0.15–0.24)∗	0.14	(0.10–0.19)∗
Treatment						
Individual-based CBT	—b		—b		—b	
Group-based CBT	0.17	(–0.01–0.35)	0.18	(–0.01–0.38)	0.06	(–0.18–0.31)
Guided self-help CBT	–0.02	(–0.27–0.23)	0.02	(–0.23–0.28)	–0.24	(–0.70–0.23)
Gender	0.09	(0.02–0.16)	0.08	(0.00–0.16)	0.10	(0.01–0.19)
Age	0.01	(–0.01–0.02)	0.01	(–0.01–0.03)	0.01	(–0.03–0.02)
Primary diagnosis						
GAD	—b		—b		—b	
SoAD	0.44	(0.34–0.53)∗	0.48	(0.37–0.59)∗	0.39	(0.27–0.51)∗
SP	0.13	(0.01–0.26)	0.22	(0.07–0.36)∗	0.02	(–0.14–0.18)
SAD	0.10	(0.00–0.20)	0.13	(0.01–0.24)	0.08	(–0.05–0.21)
Other	–0.18	(–0.32–0.03)	–0.17	(–0.33–0.00)	–0.16	(–0.34–0.03)
Secondary analyses^c^						
Comorbid externalizing disorder	0.16	(0.06–0.27)∗	0.23	(0.11–0.34)∗	0.11	(–0.02–0.24)
Comorbid mood disorder	0.19	(0.06–0.32)∗	0.23	(0.08–0.37)∗	0.15	(–0.02–0.31)
Parental psychopathology	0.06	(0.02–0.10)∗	0.04	(–0.01–0.09)	0.09	(0.03–0.14)∗

Note: All models included the random effects of trial. Regression weights (β) significantly greater than 0 indicate that this variable is associated with a poorer reduction in symptom severity after treatment. CBT = cognitive-behavioral therapy; GAD = generalized anxiety disorder; Other = other anxiety disorder; SAD = separation anxiety disorder; SoAD = social anxiety disorder; SP = specific phobia.

a To account for data collected longitudinally, these models included the random effects of participant and the linear and quadratic effects of time.

b Reference category.

c Include comorbidity and parental psychopathology.

∗ *p* < .008.

**Table 3:** Results of Logistic Mixed Models Examining Predictors of Remission (Absence of the Primary Diagnosis) Using Data From All Time Points, or Separately Using Only the Posttreatment or at Follow-Up Assessments

Characteristic	All Time Points^a^		Posttreatment Assessment		Follow-Up Assessments^a^	
	OR	(95% CI)	OR	(95% CI)	OR	(95% CI)
Severity of primary diagnosis at baseline	0.54	(0.44–0.65)∗	0.69	(0.61–0.78)∗	0.50	(0.36–0.69)∗
Treatment						
Individual-based CBT	—b		—b		—b	
Group-based CBT	0.49	(0.21–1.13)	0.60	(0.32–1.09)	0.87	(0.24–3.13)
Guided self-help CBT	0.33	(0.10–1.04)	0.47	(0.23–0.98)	1.80	(0.13–25.57)
Gender	0.76	(0.55–1.05)	0.84	(0.67–1.05)	0.78	(0.47–1.29)
Age	1.00	(0.92–1.08)	0.98	(0.93–1.04)	1.05	(0.92–1.19)
Primary diagnosis						
GAD	—b		—b		—b	
SoAD	0.18	(0.11–0.28)∗	0.31	(0.23–0.42)∗	0.18	(0.08–0.39)∗
SP	0.59	(0.33–1.04)	0.58	(0.40–0.86)∗	0.90	(0.37–2.17)
SAD	0.76	(0.47–1.21)	0.70	(0.51–0.96)	1.02	(0.50–2.12)
Other	1.99	(0.99–3.99)	1.55	(0.95–2.53)	1.76	(0.60–5.16)
Secondary analyses^c^						
Comorbid externalizing disorder	0.57	(0.35–0.94)	0.66	(0.48–0.91)	0.70	(0.35–1.43)
Comorbid mood disorder	0.43	(0.23–0.80)∗	0.58	(0.39–0.87)	0.43	(0.17–1.06)
Parental psychopathology	0.80	(0.65–0.98)	0.91	(0.79–1.04)	0.71	(0.52–0.97)

Note: All models included the random effects of trial. Regression weights (β) significantly greater than 0 indicate that this variable is associated with a poorer reduction in symptom severity after treatment. CBT = cognitive-behavioral therapy; GAD = generalized anxiety disorder; Other = other anxiety disorder; OR = odds ratio; SAD = separation anxiety disorder; SoAD = social anxiety disorder; SP = specific phobia.

a To account for data collected longitudinally, these models included the random effects of participant and the linear and quadratic effects of time.

b Reference category.

c Includes comorbidity and parental psychopathology.

∗ *p* < .008.
