# Supplementary material for: Clinical Predictors of Response to Cognitive-Behavioral Therapy in Pediatric Anxiety Disorders: The Genes for Treatment (GxT) Study
Source: J Am Acad Child Adolesc Psychiatry. 2015 Jun;54(6):454–63. doi: 10.1016/j.jaac.2015.03.018 (PMC4469376; doi:10.1016/j.jaac.2015.03.018)
Supplement: Supplemental Material [file mmc1.docx]

**SUPPLEMENTARY MATERIAL**

**Method**

Unless otherwise specified, clinical trials included all primary anxiety disorder diagnoses. All sites made secondary anxiety disorder diagnoses where appropriate. According to the categories determined by Manassis et al.,[^1^](#_ENREF_1) the first author coded the type of parental involvement in the treatment offered at each site, confirmed by the clinical leads from each site. The type of parental involvement was coded into the following three categories: 1) low parental involvement: parents involved in less than 50% of sessions or for a brief time at the beginning of each session; 2) active parental involvement + low contingency management (CM)/transfer of control (TC): parents attended more than 50% of sessions, but parent involvement did not involve a focus on CM or TC. For example, parental involvement focused on parent/child interactions or parent anxiety or parenting but did not use contingency management or a model by which the therapist gradually transferred control to the parent; 3) active parental involvement  + high CM/TC: parents attended more than 50% of sessions and included a focus on CM or TC (e.g., parental involvement focused on teaching parents to reward children for facing anxiety-provoking situations and/or the therapy used a model by which the  therapist gradually transferred control to the parent.

**Sydney, Australia** (n **=** 706). Participants aged 6–18 were recruited from the Centre for Emotional Health, Macquarie University, Sydney. All participants completed the Cool Kids Program,[^2^](#_ENREF_2) with 9–12 family sessions involving the parents (the majority of which were conducted in groups; 8% of the sample’s DNA were collected retrospectively). Variations on this treatment program include a subgroup from previous randomized trials who received group, individual, or phone-based cognitive-behavioral therapy (CBT) sessions[^3^](#_ENREF_3)^,^[^4^](#_ENREF_4); participants from a guided self-help trial with phone support for children in rural Australia [^5^](#_ENREF_5); a group from a trial with additional parental anxiety management[^6^](#_ENREF_6); and those recruited from an ongoing randomized trial of progressive allocation to treatment (stepped care). Therapists from these trials included a mix of clinical psychology master’s students and experienced therapists. In the stepped care trial, guided self-help was led by four year trained psychologists, and the final step was delivered by experienced clinicians. Otherwise, therapy was delivered by clinical psychology graduate students. Treatments were coded as active parental involvement with high CM/TC.

**Reading** (n = 340) **and Oxford** (n = 21), **UK** Participants aged 5–18 were recruited jointly from Reading and Oxford from eight trials at the Berkshire Child Anxiety Clinic (University of Reading) and the Oxfordshire Primary Child and Adolescent Mental Health Service. Participants received treatment in three main themes; one focusing on children with anxious mothers; a set of trials using a parent-guided self-help CBT program; and an online CBT program for adolescents.

*The Mother and Child (MaCh) project.* [^7^](#_ENREF_7) Children whose mother also had a current anxiety disorder completed an 8-session manual-based CBT treatment based on the Cool Kids Program.[^8^](#_ENREF_8) The mothers of these children also received extra sessions focusing on their own anxiety and on mother–child interactions. Therapists in this trial had several years of experience.

*Overcoming.* Children were treated with a parent-guided self-help CBT program, comprised of the same primary components as the Cool Kids Program.[^8^](#_ENREF_8)^,^[^9^](#_ENREF_9) This consisted of 2–4 in-person sessions and 2–4 telephone sessions. A subset of this group with a primary anxiety disorder diagnosis of social phobia also received targeted cognitive bias modification training (CBM-I, [^10^](#_ENREF_10)). Additionally, participants with highly anxious parents (screened using the Depression Anxiety Stress Scales [DASS] or by meeting Anxiety Disorders Interview Schedule [ADIS] criteria) were randomized to groups in a trial including additional sessions for the parents, which focused on strategies for tolerating children’s negative emotions. In Oxford, treatment was based on the same basic program and delivered by primary health workers as part of a feasibility trial.[^11^](#_ENREF_11)

*BRAVE*. The final treatment group completed a therapist-supported online CBT program for adolescents (BRAVE), consisting of 10 sessions, half with 5 additional parent sessions and half without parent sessions. Therapists from these trials included a mix of clinical psychology master’s students and experienced therapists

**Aarhus, Denmark** (n = 124). Participants aged 7-17 years were recruited from the Department of Psychology and Behavioral Sciences, Aarhus University, and all anxiety disorder diagnoses were included. Participants received CBT using the Cool Kids manual (including the adolescent version where appropriate [^8^](#_ENREF_8)^,^[^12^](#_ENREF_12)). Participants came from two groups: one aged 7–17, from a trial including treatment and waitlist conditions; and another group aged 7–12 from a trial comparing efficacy of traditional group-based treatment with Cool Kids versus a guided self-help version with clinician support (bibliotherapy). In both trials only participants that received in-person CBT were included. Therapists from these trials included a mix of novice and experienced therapists. Treatments were coded as active parental involvement with high CM/TC.

**Bergen, Norway** (n = 119). Participants aged 5-13 were recruited from the child part of the “Assessment and Treatment – Anxiety in Children and Adults” study, Haukeland University Hospital, Bergen. Patients referred to outpatient mental health clinics in Western Norway, with a primary diagnosis of separation anxiety, social phobia, or generalized anxiety, received group or individual treatment with the FRIENDS program (4^th^ edition [^13^](#_ENREF_13)^,^[^14^](#_ENREF_14)) in a randomized controlled trial comparing active treatment with a waitlist condition.[^15^](#_ENREF_15) Therapists from these trials included a mix of novice and experienced therapists. All therapists were experienced in working with children. Treatments were coded as having low parental involvement.

**Bochum, Germany** (n = 57). Participants aged 5–18 were recruited from the Research and Treatment Centre for Mental Health, Ruhr-Universität Bochum. Participants received either exposure-based CBT (8–25 sessions, with sessions occurring at least every 2 weeks (coded as low parental involvement, the Coping Cat program [^16^](#_ENREF_16) coded as low parental involvement, or a family-based version of CBT specifically designed to target separation anxiety disorder coded as active parental involvement with high CM/TC [TAFF [^17^](#_ENREF_17)^,^[^18^](#_ENREF_18)]). Diagnoses were provided separately for parent and child report. The primary diagnosis was selected as being the most severe from either reporter. If the most severe disorder reported by each was of equal severity but was a different diagnosis, the parent-reported diagnosis was selected. Therapists from this site included a mix of novice and experienced therapists. All therapists had a master’s degree in Clinical Psychology and were in an advanced state of child CBT training or were licensed child CBT therapists.

**Basel, Switzerland** (n = 49). Participants aged 5–13 (all with a primary diagnosis of separation anxiety disorder) were recruited from the faculty of psychology, University of Basel. All participants took part in a randomized controlled trial comparing a family-based version of CBT specifically designed to target separation anxiety disorder coded as active parental involvement with high CM/TC (TAFF[^17^](#_ENREF_17)^,^[^18^](#_ENREF_18)) with Coping Cat coded as low parental involvement.[^16^](#_ENREF_16) All participants received 16 sessions over 12 weeks. Therapists from this site included a mix of novice and experienced therapists. All therapists had a master’s degree in Clinical Psychology and were in an advanced state of child CBT training or were licensed child CBT therapists.

**Groningen, the Netherlands** (n = 37). Participants aged 8 to 17 were recruited from the Department of Child and Adolescent Psychiatry, University of Groningen. All participants were treated within a randomized controlled trial of Coping Cat (Dutch version),[^19^](#_ENREF_19) including 12 individual child sessions and 2 parent sessions. Treatment was coded as low parental involvement. Therapists conducting the treatment were a mix of novice and experienced therapists.

**Florida, USA** (n = 50). Participants aged 7–16 (including all primary anxiety disorder diagnoses except posttraumatic stress disorder [PTSD]) were recruited from the Child Anxiety and Phobia Program, Florida International University, Miami. All participants received 12–14 hour-long sessions of individual manualized CBT. Additionally, two conditions included parental involvement focusing on different parent skills (Relationship Skills Training coded as active parental involvement with low CM/TC or Reinforcement Skills Training coded as active parental involvement with high contingency management/transfer of control). Therapists from these trials included a mix of novice and experienced therapists.

**Cambridge, UK** (n = 12). Participants aged 8–17 were recruited from the Medical Research Council Cognition and Brain Sciences Unit, Cambridge, UK. Participants were taking part in the Acute Stress Programme for Children and Teenagers (ASPECTS) trial, which recruited individuals exposed to a recent (i.e. in the previous six months) traumatic stressor (i.e. any event that involve the threat of death, severe injury, or threat to bodily integrity, or witnessing such an event). Those that developed PTSD were randomized to a 10-week waitlist or individual PTSD-specific CBT,[^20^](#_ENREF_20) which consisted of up to 10 sessions over a 10-week period. Only participants that received treatment were included. Treatment was coded as active parental involvement with low CM/TC. The two therapists delivering treatment were experienced.

**Amsterdam, the Netherlands** (n = 4). Participants aged 10–14 were recruited from the Academic Treatment Centre for Parent and Child, University of Amsterdam (UvA) Minds and received either 12 weeks of CBT in individual sessions or 8 weeks of CBT in group sessions, according to the Dutch protocol “Discussing + Doing = Daring.”^20^ Treatment was coded as low parental involvement. Diagnoses were provided separately for parent and child report, with the primary diagnosis selected from these data by the trial manager. Therapists in this trial had several years of experience.

**Table S1** Results of Linear and Logistic Mixed Models Examining Predictors of Treatment Response (Change in Severity of the Primary Diagnosis From Baseline) and Remission (Absence of the Primary Diagnosis), Respectively

|  | Response | | | Remission | | |
| --- | --- | --- | --- | --- | --- | --- |
|  | All Time Points^a^ | Posttreatment Assessment | Follow-Up Assessments^a^ | All Time Points^a^ | Posttreatment Assessment | Follow-Up Assessments^a^ |
|  | β (95%CI) | β (95%CI) | β (95%CI) | OR (95%CI) | OR (95%CI) | OR (95%CI) |
| Severity of primary diagnosis at baseline | 0.29 (0.27-0.31)* | 0.29 (0.26-0.32)* | 0.13 (0.08-0.18)* | 0.57 (0.48-0.69)* | 0.72 (0.64-0.82)* | 0.54 (0.39-0.74) |
| Treatment |  |  |  |  |  |  |
| Individual-based CBT | - ^b^ | - ^b^ | - ^b^ | - ^b^ | - ^b^ | - ^b^ |
| Group-based CBT | 0.10 (0.01-0.19) | 0.09 (-0.03-0.22) | 0.04 (-0.20-0.28) | 0.53 (0.23-1.21) | 0.65 (0.37-1.15) | 0.94 (0.26-3.38) |
| Guided self-help CBT | 0.01 (-0.11-0.10) | -0.02 (-0.16-0.12) | -0.28 (-0.75-0.18) | 0.38 (0.12-1.16) | 0.52 (0.26-1.05) | 2.51 (0.19-33.81) |
| Sex | 0.04 (-0.00-0.08) | 0.03 (-0.03-0.09) | 0.09 (0.00-0.18) | 0.80 (0.58-1.11) | 0.87 (0.70-1.09) | 0.80 (0.48-1.34) |
| Age | 0.01 (-0.01-0.01) | 0.01 (-0.01-0.02) | 0.01 (-0.03-0.02) | 1.00 (0.92-1.09) | 0.99 (0.94-1.05) | 1.03 (0.91-1.17) |
| Presence of SoAD | 0.12 (0.07-0.16)* | 0.13 (0.07-0.20)* | 0.24 (0.15-0.34)* | 0.29 (0.20-0.42)* | 0.50 (0.39-0.63)* | 0.23 (0.12-0.45)* |
| **Note:** Analyses were conducted using data from all time points, or separately using only the posttreatment or follow-up assessments. These analyses consider the effects of a diagnosis of social anxiety disorder (SoAD) anywhere in the child’s diagnostic profile on outcomes. CBT = cognitive-behavioral therapy.  ^a^ To account for data collected longitudinally, these models included the random effects of participant and the linear and quadratic effects of time. All models included the random effects of trial. Regression weights (β) significantly greater than zero indicate that this variable is associated with a poorer reduction in symptom severity following treatment.  ^b^ Reference category.  * *p*<.008 | | | | | | |

| **Table S2.** Results of Linear and Logistic Mixed Models Examining Predictors of Response (Change in Severity of the Primary Diagnosis From Baseline) and Remission (Absence of the Primary Diagnosis), Respectively | | | | | | | |
| --- | --- | --- | --- | --- | --- | --- | --- |
|  | Response | | | Remission | | | |
|  | All Time Points^a^ | Posttreatment Assessment | Follow-Up Assessments^a^ | All Time Points^a^ | Posttreatment Assessment | | Follow-Up Assessments^a^ |
|  | β (95%CI) | β (95%CI) | β (95%CI) | OR (95%CI) | OR (95%CI) | | OR (95%CI) |
| Severity of Primary Diagnosis at Baseline | 0.15 (0.11-0.19)* | 0.16 (0.11-0.21)* | 0.14 (0.08-0.20)* | 0.58 (0.47-0.73)* | 0.76 (0.65-0.87)* | | 0.50 (0.34-0.73)* |
| Treatment |  |  |  |  |  | |  |
| Individual-based CBT | - ^b^ | - ^b^ | - ^b^ | - ^b^ | - ^b^ | | - ^b^ |
| Group-based CBT | 0.41 (0.17-0.65) | 0.44 (0.21-0.66) | 0.20 (-0.19-0.58) | 0.25 (0.07-0.89) | 0.42 (0.20-0.89) | | 0.65 (0.05-8.63) |
| Guided self-help CBT | 0.12 (-0.12-0.36) | 0.19 (-0.04-0.42) | -0.15 (-0.59-0.28) | 0.33 (0.09-1.21) | 0.48 (0.23-1.01) | | 1.65 (0.09-31.70) |
| Sex | 0.11 (0.03-0.19) | 0.11 (0.02-0.20) | 0.11 (0.02-0.21) | 0.69 (0.47-1.01) | 0.81 (0.63-1.04) | | 0.71 (0.41-1.24) |
| Age | 0.01 (-0.02-0.02) | 0.01 (-0.01-0.03) | -0.02 (-0.05-0.00) | 1.04 (0.94-1.15) | 1.00 (0.93-1.06) | | 1.16 (1.00-1.35) |
| Primary diagnosis | | | |  | |  | |
| GAD | - ^b^ | - ^b^ | - ^b^ | - ^b^ | - ^b^ | | - ^b^ |
| SoAD | 0.47 (0.37-0.58)* | 0.50 (0.38-0.63)* | 0.44 (0.30-0.57)* | 0.14 (0.08-0.24)* | 0.28 (0.20-0.39)* | | 0.13 (0.05-0.34)* |
| SP | 0.16 (0.03-0.29) | 0.25 (0.09-0.40)* | 0.05 (-0.11-0.22) | 0.47 (0.25-0.87) | 0.53 (0.35-0.80) | | 0.64 (0.25-1.61) |
| SAD | 0.08 (-0.03-0.19) | 0.09 (-0.04-0.22) | 0.05 (-0.09-0.19) | 0.74 (0.43-1.28) | 0.69 (0.48-0.99) | | 1.14 (0.51-2.56) |
| Other | -0.13 (-0.28-0.03) | -0.14 (-0.32-0.04) | -0.08 (-0.28-0.11) | 1.44 (0.68-3.06) | 1.31 (0.78-2.19) | | 1.11 (0.38-3.28) |
| Secondary analyses including comorbidity and parental psychopathology | | | |  | |  | |
| Comorbid externalizing disorder | 0.16 (0.06-0.27)* | 0.23 (0.11-0.34)* | 0.11 (-0.02-0.24) | 0.57 (0.35-0.94) | 0.66 (0.48-0.91) | | 0.70 (0.35-1.43) |
| Comorbid mood disorder | 0.19 (0.06-0.32)* | 0.23 (0.08-0.37)* | 0.15 (-0.02-0.31) | 0.43 (0.23-0.80)* | 0.58 (0.39-0.87) | | 0.43 (0.17-1.06) |
| Parental psychopathology | 0.06 (0.02-0.10)* | 0.04 (-0.01-0.09) | 0.09 (0.03-0.14)* | 0.80 (0.65-0.98) | 0.91 (0.79-1.04) | | 0.71 (0.52-0.97) |
| **Note:** Analyses were conducted using data from all time points, or separately using only the posttreatment or follow-up assessments. These secondary analyses explore the effects of comorbid mood or externalizing disorders and parental psychopathology on remission and response in trials that measured these factors. CBT = cognitive-behavioral therapy; GAD = generalized anxiety disorder; Other = other anxiety disorder; SAD = separation anxiety disorder; SoAD = social anxiety disorder; SP = specific phobia.  ^a^ To account for data collected longitudinally, these models included the random effects of participant and the linear and quadratic effects of time. All models included the random effects of trial. Regression weights (β) significantly greater than zero indicate that this variable is associated with a poorer reduction in symptom severity following treatment. Odds ratios significantly greater than 1 indicate that this variable is associated with increased likelihood of remission following treatment (i.e. absence of the primary diagnosis).  ^b^ Reference category  * *p*<.008. | | | | | | | |

| **Table S3.** Results of Linear and Logistic Mixed Models Examining Predictors of Response (Change in Severity of the Primary Diagnosis From Baseline) and Remission (Absence of the Primary Diagnosis), Respectively | | | | | | | |
| --- | --- | --- | --- | --- | --- | --- | --- |
|  | Response | | | Remission | | | |
|  | All Time Points^a^ | Posttreatment Assessment | Follow-Up Assessments^a^ | All Time Points^a^ | Posttreatment Assessment | | Follow-Up Assessments^a^ |
|  | β (95%CI) | β (95%CI) | β (95%CI) | OR (95%CI) | OR (95%CI) | | OR (95%CI) |
| Severity of Primary Diagnosis at Baseline | 0.18 (0.14-0.21)* | 0.19 (0.15-0.24)* | 0.16 (0.11-0.20)* | 0.54 (0.44-0.65)* | 0.69 (0.60-0.78)* | | 0.49 (0.35-0.68)* |
| Treatment |  |  |  |  |  | |  |
| Individual-based CBT | - ^b^ | - ^b^ | - ^b^ | - ^b^ | - ^b^ | | - ^b^ |
| Group-based CBT | 0.16 (-0.03-0.35) | 0.20 (-0.02-0.41) | 0.08 (-0.17-0.32) | 0.46 (0.18-1.17) | 0.56 (0.27-1.14) | | 0.70 (0.18-2.79) |
| Guided self-help CBT | -0.05 (-0.33-0.23) | 0.04 (-0.25-0.33) | -0.22 (-0.67-0.23) | 0.31 (0.08-1.14) | 0.43 (0.18-1.04) | | 1.38 (0.11-18.03) |
| Sex | 0.09 (0.02-0.16) | 0.09 (0.00-0.17) | 0.10 (0.01-0.18) | 0.75 (0.54-1.04) | 0.83 (0.66-1.04) | | 0.77 (0.47-1.27) |
| Age | 0.01 (-0.01-0.02) | 0.01 (-0.01-0.03) | 0.01 (-0.03-0.02) | 1.00 (0.92-1.09) | 0.99 (0.93-1.05) | | 1.04 (0.91-1.19) |
| Primary diagnosis | | | |  | |  | |
| GAD | - ^b^ | - ^b^ | - ^b^ | - ^b^ | - ^b^ | | - ^b^ |
| SoAD | 0.44 (0.35-0.54)* | 0.48 (0.37-0.59)* | 0.40 (0.28-0.52)* | 0.18 (0.11-0.28)* | 0.31 (0.23-0.42)* | | 0.17 (0.08-0.38)* |
| SP | 0.14 (0.01-0.26) | 0.21 (0.07-0.36)* | 0.02 (-0.13-0.17)* | 0.57 (0.32-1.02) | 0.57 (0.39-0.85)* | | 0.86 (0.36-2.09) |
| SAD | 0.10 (0.00-0.21) | 0.13 (0.01-0.25) | 0.07 (-0.06-0.20) | 0.74 (0.47-1.19) | 0.69 (0.50-0.95) | | 1.00 (0.48-2.07) |
| Other | -0.17 (-0.32--0.03) | -0.16 (-0.32-0.01) | -0.16 (-0.34-0.01) | 1.96 (0.98-3.94) | 1.53 (0.94-2.50) | | 1.77 (0.60-5.20) |
| Parent involvement |  |  |  |  |  | |  |
| Low | - ^b^ | - ^b^ | - ^b^ | - ^b^ | - ^b^ | | - ^b^ |
| Active + Low CM/TC | -0.06 (-0.43-0.31) | -0.18 (-0.54-0.18) | 0.48 (-0.10-1.06) | 0.95 (0.17-5.36) | 1.23 (0.40-3.74) | | 0.03 (0.00-1.06) |
| Active + High CM/TC | 0.01 (-0.23-0.23) | -0.13 (-0.38-0.12) | 0.21 (-0.09-0.50) | 1.17 (0.38-3.60) | 1.31 (0.61-2.82) | | 0.39 (0.05-3.23) |
| Secondary analyses including comorbidity and parental psychopathology | | | |  | |  | |
| Comorbid externalizing disorder | 0.16 (0.06-0.26)* | 0.22 (0.11-0.34)* | 0.11 (-0.02-0.23) | 0.58 (0.36-0.94) | 0.67 (0.48-0.92) | | 0.72 (0.36-1.46) |
| Comorbid mood disorder | 0.19 (0.06-0.32)* | 0.23 (0.08-0.38)* | 0.15 (-0.02-0.31) | 0.43 (0.23-0.79)* | 0.58 (0.38-0.86)* | | 0.43 (0.18-1.06) |
| Parental psychopathology | 0.06 (0.02-0.10)* | 0.04 (-0.01-0.09) | 0.08 (0.03-0.14)* | 0.80 (0.65-0.98) | 0.91 (0.80-1.04) | | 0.72 (0.53-0.98) |
| **Note.** Analyses were conducted using data from all time points, or separately using only the posttreatment or follow-up assessments. These analyses also included *parent involvement* as a covariate. Active + High CM/TC = active involvement with emphasis on contingency management or transfer of control; Active + Low CM/TC = active involvement without emphasis on contingency management and transfer of control; CBT = cognitive-behavioral therapy; GAD = generalized anxiety disorder; Low = low parental involvement; Other = other anxiety disorder; SAD = separation anxiety disorder; SoAD = social anxiety disorder; SP = specific phobia.  ^a^ To account for data collected longitudinally, these models included the random effects of participant and the linear and quadratic effects of time. All models included the random effects of trial. Regression weights (β) significantly greater than zero indicate that this variable is associated with a poorer reduction in symptom severity following treatment. Odds ratios significantly greater than 1 indicate that this variable is associated with increased likelihood of remission following treatment (i.e. absence of the primary diagnosis).  ^b^ Reference category * *p*<.008. | | | | | | | |

| **Table S4.** Results of Linear and Logistic Mixed Models Examining Predictors of Response (Change in Severity of the Primary Diagnosis From Baseline) and Remission (Absence of the Primary Diagnosis), Respectively | | | | | | | |
| --- | --- | --- | --- | --- | --- | --- | --- |
|  | Response | | | Remission | | | |
|  | All Time Points^a^ | Posttreatment Assessment | Follow-Up Assessments^a^ | All Time Points^a^ | Posttreatment Assessment | | Follow-Up Assessments^a^ |
|  | β (95%CI) | β (95%CI) | β (95%CI) | OR (95%CI) | OR (95%CI) | | OR (95%CI) |
| Severity of primary diagnosis at baseline | 0.18 (0.14-0.21)* | 0.20 (0.15-0.24)* | 0.15 (0.11-0.20)* | 0.54 (0.45-0.65)* | 0.69 (0.61-0.78)* | | 0.50 (0.36-0.69)* |
| Treatment |  |  |  |  |  | |  |
| Individual-based CBT | - ^b^ | - ^b^ | - ^b^ | - ^b^ | - ^b^ | | - ^b^ |
| Group-based CBT | 0.11 (-0.07-0.29) | 0.11 (-0.10-0.31) | 0.06 (-0.18-0.30) | 0.62 (0.27-1.43) | 0.76 (0.42-1.37) | | 0.83 (0.23-3.09) |
| Guided self-help CBT | -0.25 (-0.55-0.05) | -0.23 (-0.55-0.09) | -0.29 (-0.81-0.23) | 0.91 (0.23-3.61) | 1.07 (0.44-2.62) | | 1.32 (0.05-32.69) |
| Sex | 0.09 (0.02-0.16) | 0.08 (0.00-0.17) | 0.09 (0.01-0.18) | 0.76 (0.55-1.05) | 0.83 (0.67-1.05) | | 0.78 (0.47-1.28) |
| Age | 0.01 (-0.01-0.02) | 0.01 (-0.01-0.04) | -0.01 (-0.03-0.02) | 0.99 (0.91-1.08) | 0.98 (0.93-1.04) | | 1.05 (0.92-1.20) |
| Primary diagnosis | | | |  | |  | |
| GAD | - ^b^ | - ^b^ | - ^b^ | - ^b^ | - ^b^ | | - ^b^ |
| SoAD | 0.44 (0.35-0.54)* | 0.48 (0.37-0.59)* | 0.39 (0.28-0.51)* | 0.18 (0.11-0.28)* | 0.31 (0.22-0.41)* | | 0.18 (0.08-0.39)* |
| SP | 0.14 (0.02-0.26) | 0.22 (0.08-0.37)* | 0.01 (-0.14-0.16) | 0.57 (0.32-1.01) | 0.57 (0.38-0.83) | | 0.90 (0.38-2.18) |
| SAD | 0.12 (0.01-0.22) | 0.14 (0.02-0.26) | 0.08 (-0.05-0.20) | 0.72 (0.45-1.15) | 0.67 (0.49-0.93) | | 1.03 (0.50-2.14) |
| Other | -0.18 (-0.33--0.04) | -0.17 (-0.34--0.01) | -0.16 (-0.34-0.02) | 2.03 (1.01-4.07) | 1.59 (0.98-2.59) | | 1.76 (0.60-5.17) |
| Number of sessions | -0.06 (-0.10--0.01) | -0.06 (-0.10--0.01) | -0.02 (-0.09-0.05) | 1.28 (1.03-1.57) | 1.21 (1.05-1.38) | | 0.91 (0.55-1.51) |
| Secondary analyses including comorbidity and parental psychopathology | | | |  | |  | |
| Comorbid externalizing disorder | 0.16 (0.06-0.26)* | 0.23 (0.11-0.34)* | 0.11 (-0.02-0.24) | 0.58 (0.35-0.94) | 0.66 (0.48-0.92) | | 0.71 (0.35-1.44) |
| Comorbid mood disorder | 0.19 (0.06-0.32)* | 0.23 (0.08-0.37)* | 0.15 (-0.02-0.31) | 0.43 (0.24-0.80)* | 0.58 (0.39-0.87)* | | 0.43 (0.17-1.07) |
| Parental psychopathology | 0.06 (0.02-0.10)* | 0.04 (-0.01-0.09) | 0.09 (0.03-0.14)* | 0.80 (0.65-0.98) | 0.91 (0.80-1.04) | | 0.72 (0.53-0.97) |
| Note. Analyses were conducted using data from all time points, or separately using only the posttreatment or follow-up assessments. These analyses also included number of sessions as a covariate. CBT = cognitive-behavioral therapy; GAD = generalized anxiety disorder; Other = other anxiety disorder; SAD = separation anxiety disorder; SoAD = social anxiety disorder; SP = specific phobia.  ^a^ To account for data collected longitudinally, these models included the random effects of participant and the linear and quadratic effects of time. All models included the random effects of trial. Regression weights (β) significantly greater than zero indicate that this variable is associated with a poorer reduction in symptom severity following treatment. Odds ratios significantly greater than 1 indicate that this variable is associated with increased likelihood of remission following treatment (i.e. absence of the primary diagnosis).  ^b^ Reference category.  * *p*<.008. | | | | | | | |

**Table S5** Results of Linear and Logistic Mixed Models Examining Predictors of Treatment Response (Change in Severity of the Primary Diagnosis From Baseline) and Remission (Absence of the Primary Diagnosis), Respectively

|  | Response | | | Remission | | | | |  |
| --- | --- | --- | --- | --- | --- | --- | --- | --- | --- |
|  | All Time Points^a^ | Posttreatment Assessment | Follow-Up Assessments^a^ | All Time Points^a^ | Posttreatment Assessment | | Follow-Up Assessments^a^ | |  |
|  | β (95%CI) | β (95%CI) | β (95%CI) | OR (95%CI) | OR (95%CI) | | OR (95%CI) | |  |
| Severity of primary diagnosis at baseline | 0.17 (0.13-0.22)* | 0.19 (0.14-0.24)* | 0.15 (0.09-0.21)* | 0.56 (0.46-0.69) | 0.71 (0.62-0.82) | | 0.51 (0.34-0.74) | |  |
| Treatment |  |  |  |  |  | |  | |  |
| Individual-based CBT | - ^b^ | - ^b^ | - ^b^ | - ^b^ | - ^b^ | | - ^b^ | |  |
| Group-based CBT | 0.16 (-0.02-0.34) | 0.17 (-0.03-0.37) | 0.05 (-0.20-0.30) | 0.54 (0.24-1.23) | 0.63 (0.34-1.16) | | 0.90 (0.26-3.14) | |  |
| Guided self-help CBT | 0.01 (-0.25-0.25) | 0.04 (-0.21-0.29) | -0.20 (-0.70-0.31) | 0.33 (0.11-1.03) | 0.47 (0.22-0.98) | | 2.29 (0.17-31.61) | |  |
| Sex | 0.07 (-0.01-0.15) | 0.07 (-0.02-0.17) | 0.07 (-0.04-0.19) | 0.82 (0.57-1.19) | 0.85 (0.65-1.10) | | 0.96 (0.53-1.76) | |  |
| Age | 0.01 (-0.01-0.03) | 0.02 (-0.01-0.04) | 0.01 (-0.02-0.03) | 0.98 (0.89-1.07) | 0.97 (0.91-1.04) | | 1.02 (0.88-1.20) | |  |
| Primary diagnosis | | | |  | |  | | | |
| GAD | - ^b^ | - ^b^ | - ^b^ | - ^b^ | - ^b^ | | - ^b^ | |  |
| SoAD | 0.45 (0.34-0.56)* | 0.48 (0.35-0.62)* | 0.40 (0.25-0.56)* | 0.22 (0.13-0.37) | 0.33 (0.23-0.48) | | 0.23 (0.09-0.59) | |  |
| SP | 0.11 (-0.03-0.26) | 0.19 (0.03-0.36) | -0.01 (-0.21-0.18) | 0.66 (0.34-1.26) | 0.62 (0.39-0.98) | | 1.07 (0.36-3.11) | |  |
| SAD | 0.09 (-0.03-0.21) | 0.12 (-0.01-0.26) | 0.04 (-0.12-0.21) | 0.92 (0.54-1.58) | 0.75 (0.52-1.10) | | 1.53 (0.62-3.79) | |  |
| Other | -0.17 (-0.34-0.00) | -0.16 (-0.36-0.04) | -0.14 (-0.38-0.09) | 2.12 (0.94-4.79) | 1.54 (0.86-2.76) | | 2.09 (0.53-8.19) | |  |
| Secondary analyses including comorbidity and parental psychopathology | | | |  | |  | | | |
| Comorbid externalizing disorder | 0.14 (0.02-0.27) | 0.27 (0.12-0.41)* | 0.01 (-0.16-0.18) | 0.61 (0.33-1.10) | 0.58 (0.39-0.86)* | | | 1.37 (0.53-3.53) |  |
| Comorbid mood disorder | 0.20 (0.05-0.35) | 0.20 (0.03-0.37) | 0.18 (-0.02-0.38) | 0.57 (0.28-1.16) | 0.69 (0.43-1.10) | | | 0.64 (0.22-1.91) |  |
| Parental psychopathology | 0.05 (-0.01-0.10) | 0.03 (-0.02-0.09) | 0.07 (-0.00-0.14) | 0.80 (0.62-1.02) | 0.89 (0.76-1.05) | | | 0.76 (0.50-1.15) |  |

Note. Analyses were conducted using data from all time points, or separately using only the posttreatment or follow-up assessments. These analyses exclude the subset (n=384) of the Genes for Treatment (GxT) sample included in previous analyses.[^21^](#_ENREF_21) CBT = cognitive-behavioral therapy; GAD = generalized anxiety disorder; Other = other anxiety disorder; SAD = separation anxiety disorder; SoAD = social anxiety disorder; SP = specific phobia.

^a^ To account for data collected longitudinally, these models included the random effects of participant and the linear and quadratic effects of time. All models included the random effects of trial. Regression weights (β) significantly greater than zero indicate that this variable is associated with a poorer reduction in symptom severity following treatment. Odds ratios significantly greater than 1 indicate that this variable is associated with increased likelihood of remission following treatment (i.e. absence of the primary diagnosis).

^b^ Reference category * *p*<.008.

**Table S6** Results of Logistic Mixed Models Examining Predictors of Remission From all Anxiety Disorders (Absence of any Anxiety Diagnosis)

|  | Remission From all Anxiety Diagnoses | | |
| --- | --- | --- | --- |
|  | All Time Points^a^ | Posttreatment Assessment | Follow-Up Assessments^a^ |
|  | β (95%CI) | β (95%CI) | β (95%CI) |
| Severity of primary diagnosis at baseline | 0.52 (0.44-0.62)* | 0.69 (0.61-0.78)* | 0.44 (0.31-0.61)* |
| Treatment |  |  |  |
| Individual-based CBT | - ^b^ | - ^b^ | - ^b^ |
| Group-based CBT | 0.98 (0.40-2.40) | 0.71 (0.36-1.40) | 1.50 (0.44-5.15) |
| Guided self-help CBT | 0.89 (0.24-3.29) | 0.73 (0.32-1.68) | 2.76 (0.36-20.94) |
| Sex | 0.70 (0.51-0.95) | 0.79 (0.63-1.00) | 0.71 (0.45-1.14) |
| Age | 1.02 (0.94-1.10) | 1.03 (0.97-1.09) | 1.00 (0.88-1.12) |
| Primary diagnosis | | | |
| GAD | - ^b^ | - ^b^ | - ^b^ |
| SoAD | 0.47 (0.31-0.71)* | 0.59 (0.43-0.81)* | 0.43 (0.22-0.83)* |
| SP | 1.57 (0.92-2.67) | 1.17 (0.79-1.72) | 2.16 (0.93-4.99) |
| SAD | 0.98 (0.63-1.53) | 0.98 (0.70-1.36) | 0.93 (0.47-1.83) |
| Other | 0.75 (0.40-1.41) | 0.90 (0.56-1.43) | 0.59 (0.23-1.55) |
| Secondary analyses including comorbidity and parental psychopathology | | | |
| Comorbid externalizing disorder | 0.53 (0.34-0.84)* | 0.58 (0.41-0.82)* | 0.68 (0.35-1.33) |
| Comorbid mood disorder | 0.50 (0.28-0.90) | 0.55 (0.35-0.87) | 0.52 (0.22-1.27) |
| Parental psychopathology | 0.78 (0.65-0.94) | 0.86 (0.75-1.00) | 0.74 (0.56-1.00) |

**Note.** Analyses were conducted using data from all time points, or separately using only the posttreatment or follow-up assessments. CBT = cognitive-behavioral therapy; GAD = generalized anxiety disorder; Other = other anxiety disorder; SAD = separation anxiety disorder; SoAD = social anxiety disorder; SP = specific phobia.

^a^ To account for data collected longitudinally, these models included the random effects of participant and the linear and quadratic effects of time. All models included the random effects of trial. Odds ratios significantly greater than 1 indicate that this variable is associated with increased likelihood of remission following treatment (i.e. absence of the primary diagnosis).

^b^ Reference category.

* *p*<.008.

**Table S7.** Results of a Linear Regression of Treatment Response (Change in Severity of the Primary Diagnosis) at Each Time Point in the Study

|  | Posttreatment  (n=1,466) | | | 3-Month Follow-Up  (n=234) | | | | 6-Month Follow-Up  (n=741) | | | 12-Month Follow-Up  (n=337) | | |
| --- | --- | --- | --- | --- | --- | --- | --- | --- | --- | --- | --- | --- | --- |
|  | β | 95%CI | *p* | β | 95%CI | | *p* | β | 95%CI | *p* | β | 95%CI | *p* |
| Severity of primary diagnosis at baseline | 0.49 | 0.38-0.59 | <.001* | 0.16 | -0.10-0.43 | | .222 | 0.48 | 0.32-0.65 | <.001* | 0.38 | 0.14-0.61 | .002* |
| Treatment |  |  |  |  |  | |  |  |  |  |  |  |  |
| Individual-based CBT | - ^a^ | - | - | - ^a^ | - | | - |  |  |  | - ^a^ | - | - |
| Group-based CBT | -0.18 | -0.86-0.50 | .599 | 0.80 | -2.33-3.94 | | .613 | 1.58 | -2.17-5.33 | .408 | -0.03 | -0.84-0.78 | .941 |
| Guided self-help CBT | -0.23 | -1.13-0.68 | .623 | - ^b^ | - | | - | 0.93 | -0.01-1.88 | .053 | - ^b^ | - | - |
| Sex | 0.22 | 0.02-0.42 | .034* | 0.43 | -0.14-1.00 | | .137 | 0.22 | -0.05-0.49 | .109 | 0.13 | -0.34-0.60 | .577 |
| Age | 0.03 | -0.02-0.09 | .237 | 0.04 | -0.09-0.17 | | .525 | -0.06 | -0.14-0.01 | .109 | 0.05 | -0.08-0.18 | .469 |
| Primary diagnosis |  |  |  |  |  | |  |  |  |  |  |  |  |
| GAD | - ^a^ | - | - | - ^a^ | - | | - |  |  |  | - ^a^ | - | - |
| SoAD | 1.21 | 0.93-1.49 | <.001* | 1.41 | 0.58-2.24 | | .001* | 0.90 | 0.54-1.26 | <.001* | 0.97 | 0.31-1.63 | .004* |
| SP | 0.58 | 0.22-0.95 | .002* | 0.62 | -0.33-1.56 | | .203 | -0.07 | -0.52-0.38 | .761 | 0.04 | -0.85-0.94 | .922 |
| SAD | 0.34 | 0.04-0.64 | .026* | 0.56 | -0.27-1.39 | | .183 | 0.03 | -0.38-0.44 | .894 | 0.37 | -0.30-1.03 | .280 |
| Other | -0.39 | -0.82-0.04 | .076 | 0.03 | -1.04-1.11 | | .950 | -0.3 | -0.85-0.25 | .288 | -0.99 | -2.10-0.12 | .081 |
| Secondary analyses including comorbidity and parental psychopathology | | | | | | | |  |  |  |  |  |  |
| Comorbid externalizing disorder | 0.54 | 0.24-0.84 | <.001* | 1.03 | | -0.09-2.15 | .070 | 0.24 | -0.11-0.58 | .183 | -0.27 | -1.00-0.46 | .458 |
| Comorbid mood disorder | 0.55 | 0.17-0.92 | .004* | 0.77 | | -0.49-2.03 | .232 | 0.33 | -0.14-0.80 | .170 | 0.48 | -0.47-1.43 | .320 |
| Parental psychopathology | 0.09 | -0.04-0.21 | .161 | 0.11 | | -0.33-0.55 | .616 | 0.16 | 0.01-0.31 | .031* | 0.64 | 0.33-0.95 | <.001* |

Note. All models also included trial as a covariate. Regression weights (β) significantly greater than zero indicate that this variable is associated with a poorer response (less change in symptom severity) at the specified time point. CBT = cognitive-behavioral therapy; GAD = generalized anxiety disorder; Other = other anxiety disorder; SAD = separation anxiety disorder; SoAD = social anxiety disorder; SP = specific phobia.

^a^ Reference category.

^b^ Data not available.

* *p* < .05

**Table S8.** Results of a Logistic Regression of Remission (Absence of the Primary Diagnosis) at Each Time Point in the Study

|  | Posttreatment  (n=1,466) | | | 3-Month Follow-Up  (n=234) | | | 6-Month Follow-Up  (n=741) | | | 12-Month Follow-Up  (n=337) | | |
| --- | --- | --- | --- | --- | --- | --- | --- | --- | --- | --- | --- | --- |
|  | OR | 95%CI | *p* | OR | 95%CI | *p* | OR | 95%CI | *p* | OR | 95%CI | *p* |
| Severity of primary diagnosis at baseline | 0.69 | 0.60-0.78 | <.001* | 0.78 | 0.57-1.06 | .108 | 0.61 | 0.50-0.76 | <.001* | 0.73 | 0.56-0.97 | .027* |
| Treatment |  |  |  |  |  |  |  |  |  |  |  |  |
| Individual-based CBT | - ^a^ | - | - | - ^a^ | - | - |  |  |  | - ^a^ | - | - |
| Group-based CBT | 1.31 | 0.58-2.95 | .522 | 1.20 | 0.06-24.92 | .906 | 1.18 | 0.10-13.75 | .898 | 0.91 | 0.42-2.00 | .824 |
| Guided self-help CBT | 3.09 | 1.04-9.20 | .043* | - ^b^ | - | - | 1.51 | 0.36-6.32 | .574 | - ^b^ | - | - |
| Sex | 0.84 | 0.67-1.05 | .131 | 0.75 | 0.39-1.45 | .397 | 0.84 | 0.61-1.17 | .313 | 0.96 | 0.58-1.61 | .891 |
| Age | 0.99 | 0.93-1.05 | .727 | 1.01 | 0.88-1.17 | .857 | 1.08 | 0.98-1.18 | .128 | 0.95 | 0.82-1.09 | .454 |
| Primary diagnosis |  |  |  |  |  |  |  |  |  |  |  |  |
| GAD | - ^a^ | - | - | - ^a^ | - | - |  |  |  | - ^a^ | - | - |
| Social Phobia | 0.31 | 0.22-0.41 | <.001* | 0.28 | 0.11-0.70 | .007* | 0.41 | 0.27-0.62 | <.001* | 0.49 | 0.25-0.97 | .042* |
| Specific Phobia | 0.57 | 0.39-0.85 | .005* | 0.55 | 0.18-1.66 | .291 | 0.96 | 0.56-1.65 | .876 | 1.75 | 0.56-5.49 | .340 |
| SAD | 0.67 | 0.49-0.93 | .017* | 0.78 | 0.31-2.00 | .607 | 1.21 | 0.73-2.02 | .456 | 1.10 | 0.56-2.19 | .775 |
| Other | 1.49 | 0.91-2.45 | .116 | 0.91 | 0.27-3.11 | .878 | 1.10 | 0.55-2.17 | .793 | 8.99 | 1.08-74.93 | .042 |
| Secondary analyses including comorbidity and parental psychopathology | | | | | | |  |  |  |  |  |  |
| Comorbid externalizing disorder | 0.69 | 0.50-0.95 | .025* | 0.31 | 0.10-0.97 | .044* | 0.88 | 0.58-1.34 | .559 | 1.47 | 0.56-3.83 | .433 |
| Comorbid mood disorder | 0.59 | 0.39-0.90 | .013* | 0.45 | 0.13-1.60 | .217 | 0.70 | 0.40-1.21 | .205 | 0.54 | 0.15-1.89 | .332 |
| Parental psychopathology | 0.91 | 0.80-1.05 | .196 | 0.87 | 0.52-1.45 | .585 | 0.86 | 0.72-1.03 | .106 | 0.52 | 0.33-0.83 | .006* |

Note. All models also included trial as a covariate. Odds ratios significantly greater than 1 indicate that this variable is associated with a lower likelihood of remission (i.e. primary diagnosis still met) at the at the specified time point. CBT = cognitive-behavioral therapy; GAD = generalized anxiety disorder; SAD = separation anxiety disorder.

^a^ Reference category.

^b^ Data not available.

* *p* < .05

**Table S9.** Results of Linear and Logistic Mixed Models Examining Predictors of Response (Change in Severity of the Primary Diagnosis From Baseline) and Remission (Absence of the Primary Diagnosis), Respectively

|  | Response | | | Remission | | | |  |
| --- | --- | --- | --- | --- | --- | --- | --- | --- |
|  | All Time Points^a^ | Posttreatment Assessment | Follow-Up Assessments^a^ | All Time Points^a^ | Posttreatment Assessment | | Follow-Up Assessments^a^ |  |
|  | β (95%CI) | β (95%CI) | β (95%CI) | OR (95%CI) | OR (95%CI) | | OR (95%CI) |  |
| Severity of primary diagnosis at baseline | 0.17 (0.13-0.21)* | 0.19 (0.15-0.23)* | 0.15 (0.10-0.20)* | 0.50 (0.40-0.63)* | 0.68 (0.59-0.77)* | | 0.51 (0.36-0.71)* |  |
| Treatment |  |  |  |  |  | |  |  |
| Individual-based CBT | - ^b^ | - ^b^ | - ^b^ | - ^b^ | - ^b^ | | - ^b^ |  |
| Group-based CBT | 0.18 (-0.02-0.37) | 0.2 (-0.03-0.42) | 0.04 (-0.21-0.30) | 0.42 (0.15-1.17) | 0.58 (0.31-1.11) | | 0.85 (0.21-3.42) |  |
| Guided self-help CBT | -0.01 (-0.29-0.27) | 0.04 (-0.25-0.33) | -0.32 (-0.81-0.16) | 0.28 (0.07-1.03) | 0.46 (0.21-0.99) | | 2.45 (0.16-38.40) |  |
| Sex | 0.09 (0.01-0.16) | 0.08 (-0.01-0.16) | 0.1 (0.01-0.18) | 0.76 (0.53-1.09) | 0.85 (0.68-1.07) | | 0.82 (0.49-1.37) |  |
| Age | 0.01 (-0.02-0.03) | 0.01 (-0.01-0.04) | -0.01 (-0.04-0.01) | 1.01 (0.91-1.12) | 0.99 (0.93-1.06) | | 1.09 (0.94-1.27) |  |
| Primary diagnosis | | | |  | |  | | |
| GAD | - ^b^ | - ^b^ | - ^b^ | - ^b^ | - ^b^ | | - ^b^ |  |
| SoAD | 0.42 (0.32-0.52)* | 0.46 (0.35-0.57)* | 0.38 (0.26-0.50)* | 0.15 (0.09-0.27)* | 0.32 (0.23-0.44)* | | 0.18 (0.08-0.40)* |  |
| SP | 0.16 (0.04-0.29) | 0.25 (0.11-0.40)* | 0.03 (-0.12-0.18) | 0.47 (0.25-0.90) | 0.55 (0.37-0.82)* | | 0.77 (0.31-1.90) |  |
| SAD | 0.11 (0.01-0.21) | 0.14 (0.02-0.26) | 0.07 (-0.06-0.19) | 0.65 (0.38-1.11) | 0.67 (0.48-0.92) | | 1.02 (0.48-2.14) |  |
| Other | -0.2 (-0.35--0.05) | -0.22 (-0.39--0.04) | -0.16 (-0.34-0.03) | 2.2 (0.99-4.88) | 1.76 (1.04-2.99) | | 1.66 (0.54-5.14) |  |
| Secondary analyses including comorbidity and parental psychopathology | | | |  | |  | |  |
| Comorbid externalizing disorder | 0.42 (0.32-0.52)* | 0.46 (0.35-0.57)* | 0.38 (0.26-0.50)* | 0.57 (0.35-0.94) | 0.67 (0.48-0.93) | | 0.67 (0.32-1.38) |  |
| Comorbid mood disorder | 0.16 (0.04-0.29) | 0.25 (0.11-0.40)* | 0.03 (-0.12-0.18) | 0.46 (0.24-0.87) | 0.58 (0.38-0.88) | | 0.50 (0.19-1.28) |  |
| Parental psychopathology | 0.11 (0.01-0.21) | 0.14 (0.02-0.26) | 0.07 (-0.06-0.19) | 0.79 (0.64-0.97) | 0.90 (0.78-1.03) | | 0.72 (0.52-0.98) |  |

Note. Analyses were conducted using data from all time points, or separately using only the posttreatment or follow-up assessments. These analyses include only participants aged between 5 and 13 (n=1,429). CBT = cognitive-behavioral therapy; GAD = generalized anxiety disorder; Other = other anxiety disorder; SAD = separation anxiety disorder; SoAD = social anxiety disorder; SP = specific phobia.

^a^ To account for data collected longitudinally, these models included the random effects of participant and the linear and quadratic effects of time. All models included the random effects of trial. Regression weights (β) significantly greater than zero indicate that this variable is associated with a poorer reduction in symptom severity following treatment. Odds ratios significantly greater than 1 indicate that this variable is associated with increased likelihood of remission following treatment (i.e. absence of the primary diagnosis).

^b^ Reference category

* *p*<.008.

**Table S10.** Results of Linear and Logistic Mixed Models Examining Predictors of Response (Change in Severity of the Primary Diagnosis From Baseline) and Remission (Absence of the Primary Diagnosis), Respectively

|  | Response | | | | | Remission | | | | |
| --- | --- | --- | --- | --- | --- | --- | --- | --- | --- | --- |
|  | All Time Points ^a^ | | Posttreatment Assessment | | Follow-Up Assessments ^a^ | All Time Points ^a^ | Posttreatment Assessment | | | Follow-Up Assessments ^a^ |
|  | β (95%CI) | | β (95%CI) | | β (95%CI) | OR (95%CI) | OR (95%CI) | | | OR (95%CI) |
| Severity of primary diagnosis at baseline | 0.18 (0.14-0.22)* | | 0.20 (0.15-0.24)* | | 0.16 (0.11-0.21)* | 0.49 (0.39-0.61)* | 0.69 (0.60-0.78)* | | | 0.46 (0.32-0.66)* |
| Treatment |  | |  | |  |  |  | | |  |
| Individual-based CBT | - ^b^ | | - ^b^ | | - ^b^ | - ^b^ | - ^b^ | | | - ^b^ |
| Group-based CBT | 0.18 (-0.01-0.36) | | 0.20 (-0.01-0.40) | | 0.06 (-0.18-0.29) | 0.46 (0.17-1.23) | 0.60 (0.32-1.12) | | | 0.87 (0.24-3.18) |
| Guided self-help CBT | 0.02 (-0.24-0.28) | | 0.07 (-0.20-0.34) | | -0.19 (-0.64-0.25) | 0.27 (0.07-0.98) | 0.45 (0.21-0.98) | | | 1.47 (0.12-18.42) |
| Sex | 0.09 (0.01-0.16) | | 0.08 (-0.01-0.17) | | 0.09 (0.01-0.18) | 0.76 (0.52-1.10) | 0.86 (0.68-1.09) | | | 0.79 (0.47-1.34) |
| Age | 0.01 (-0.01-0.03) | | 0.02 (-0.01-0.04) | | 0.01 (-0.03-0.02) | 0.98 (0.89-1.08) | 0.98 (0.92-1.04) | | | 1.03 (0.90-1.18) |
| Primary diagnosis | | | | | |  | | |  | |
| GAD | - ^b^ | - ^b^ | | - ^b^ | | - ^b^ | - ^b^ | | | - ^b^ |
| SoAD | 0.44 (0.34-0.53)* | 0.48 (0.37-0.59)* | | 0.39 (0.27-0.51)* | | 0.14 (0.08-0.25)* | 0.31 (0.23-0.42)* | | | 0.18 (0.08-0.39)* |
| SP | 0.13 (0.01-0.26) | 0.22 (0.07-0.36) | | 0.01 (-0.14-0.15) | | 0.53 (0.28-1.00) | 0.58 (0.40-0.86)* | | | 0.95 (0.39-2.31) |
| SAD | 0.11 (0.01-0.21) | 0.14 (0.02-0.25) | | 0.08 (-0.05-0.20) | | 0.68 (0.40-1.15) | 0.69 (0.50-0.95) | | | 1.04 (0.50-2.18) |
| Secondary analyses including comorbidity and parental psychopathology | | | | | |  | | |  | |
| Comorbid externalizing disorder | 0.18 (0.07-0.28)* | 0.24 (0.12-0.36)* | | 0.12 (-0.01-0.25) | | 0.54 (0.32-0.92) | | 0.63 (0.45-0.88) | | 0.73 (0.35-1.53) |
| Comorbid mood disorder | 0.2 (0.06-0.33)* | 0.23 (0.08-0.38)* | | 0.16 (-0.01-0.33) | | 0.43 (0.22-0.82) | | 0.61 (0.40-0.93) | | 0.36 (0.14-0.98) |
| Parental psychopathology | 0.06 (0.01-0.10) | 0.04 (-0.01-0.09) | | 0.09 (0.03-0.14)* | | 0.79 (0.64-0.98) | | 0.92 (0.80-1.05) | | 0.69 (0.50-0.96) |

**Note.** These analyses include only those with a diagnosis of generalized anxiety disorder (GAD), social anxiety disorder (SoAD), specific phobia (SP), or separation anxiety disorder (SAD) (n= 1,406). CMT = cognitive-behavioral therapy.

^a^ To account for data collected longitudinally, these models included the random effects of participant and the linear and quadratic effects of time. All models included the random effects of trial. Regression weights (β) significantly greater than zero indicate that this variable is associated with a poorer reduction in symptom severity following treatment. Odds ratios significantly greater than 1 indicate that this variable is associated with increased likelihood of remission following treatment (i.e. absence of the primary diagnosis).

^b^ Reference category.

* *p*<.008.

**Table S11.** Results of Linear and Logistic Mixed Models Examining Predictors of Response (Change in Severity of the Primary Diagnosis From Baseline) and Remission (Absence of the Primary Diagnosis), Respectively

|  | Response | | | | Remission | | | | | |
| --- | --- | --- | --- | --- | --- | --- | --- | --- | --- | --- |
|  | All Time Points^a^ | Posttreatment Assessment | Follow-Up Assessments^a^ | | All Time Points^a^ | Posttreatment Assessment | | Follow-Up Assessments^a^ | | |
|  | β (95%CI) | β (95%CI) | β (95%CI) | | OR (95%CI) | OR (95%CI) | | OR (95%CI) | | |
| Severity of primary diagnosis at baseline | 0.18 (0.14-0.22)* | 0.2 (0.16-0.25)* | 0.16 (0.11-0.21)* | | 0.51 (0.41-0.64)* | 0.68 (0.60-0.78)* | | 0.52 (0.38-0.72)* | | |
| Treatment |  |  |  | |  |  | |  | | |
| Individual-based CBT | - ^b^ | - ^b^ | - ^b^ | | - ^b^ | - ^b^ | | - ^b^ | | |
| Group-based CBT | 0.13 (-0.05-0.32) | 0.14 (-0.06-0.34) | 0.06 (-0.18-0.29) | | 0.55 (0.21-1.42) | 0.69 (0.38-1.26) | | 1.00 (0.31-3.27) | | |
| Guided self-help CBT | -0.08 (-0.34-0.18) | -0.05 (-0.30-0.21) | -0.26 (-0.67-0.16) | | 0.42 (0.12-1.48) | 0.60 (0.28-1.25) | | 2.04 (0.24-17.18) | | |
| Sex | 0.09 (0.02-0.16) | 0.08 (0.00-0.17) | 0.09 (0.00-0.18) | | 0.71 (0.49-1.02) | 0.82 (0.65-1.03) | | 0.78 (0.47-1.27) | | |
| Age | 0.01 (-0.01-0.02) | 0.01 (-0.01-0.03) | 0.01 (-0.03-0.02) | | 0.99 (0.90-1.09) | 0.99 (0.93-1.05) | | 1.02 (0.90-1.16) | | |
| Primary diagnosis | | | | |  | |  | | |  |
| GAD | - ^b^ | - ^b^ | | - ^b^ | - ^b^ | - ^b^ | | | - ^b^ | |
| SoAD | 0.45 (0.35-0.55)* | 0.50 (0.38-0.61)* | | 0.41 (0.29-0.53)* | 0.13 (0.08-0.23)* | 0.29 (0.21-0.40)* | | | 0.18 (0.08-0.39)* | |
| SP | 0.15 (0.02-0.28) | 0.24 (0.09-0.39)* | | 0.01 (-0.15-0.17) | 0.49 (0.26-0.94) | 0.57 (0.38-0.85)* | | | 0.85 (0.36-2.04) | |
| SAD | 0.11 (0.00-0.21) | 0.14 (0.03-0.26) | | 0.07 (-0.07-0.20) | 0.64 (0.38-1.09) | 0.66 (0.48-0.92) | | | 1.08 (0.53-2.21) | |
| Other | -0.17 (-0.32--0.01) | -0.15 (-0.32-0.02) | | -0.17 (-0.35-0.02) | 1.75 (0.82-3.76) | 1.40 (0.85-2.31) | | | 1.77 (0.62-5.04) | |
| Secondary analyses including comorbidity and parental psychopathology | | | | |  | |  | | |  |
| Comorbid externalizing disorder | 0.17 (0.07-0.28)* | 0.24 (0.12-0.36)* | | 0.11 (-0.03-0.24) | 0.54 (0.31-0.91) | 0.65 (0.47-0.91) | | | 0.72 (0.36-1.43) | |
| Comorbid mood disorder | 0.19 (0.06-0.33)* | 0.23 (0.08-0.38)* | | 0.16 (-0.02-0.33) | 0.41 (0.21-0.80) | 0.58 (0.38-0.88) | | | 0.42 (0.17-1.03) | |
| Parental psychopathology | 0.06 (0.02-0.10) | 0.04 (-0.01-0.09) | | 0.09 (0.04-0.15)* | 0.80 (0.64-0.99) | 0.91 (0.80-1.05) | | | 0.71 (0.53-0.96) | |

Note. These analyses include only participants who received a treatment that was not diagnosis specific (n=1,423). CBT = cognitive-behavioral therapy; GAD = generalized anxiety disorder; Other = other anxiety disorder; SAD = separation anxiety disorder; SoAD = social anxiety disorder; SP = specific phobia.

^a^ To account for data collected longitudinally, these models included the random effects of participant and the linear and quadratic effects of time. All models included the random effects of trial. Regression weights (β) significantly greater than zero indicate that this variable is associated with a poorer reduction in symptom severity following treatment. Odds ratios significantly greater than 1 indicate that this variable is associated with increased likelihood of remission following treatment (i.e. absence of the primary diagnosis).

^b^ Reference category.

* *p*<.008.

References

**1.** Manassis K, Lee TC, Bennett K, et al. Types of parental involvement in CBT with anxious youth: a preliminary meta-analysis. *Journal of consulting and clinical psychology.* 2014;82(6):1163-1172.

**2.** Rapee R, Lyneham H, Schniering C, et al. *The Cool Kids® Child and Adolescent Anxiety Program.* Sydney: Centre for Emotional Health, Macquarie University; 2006.

**3.** Hudson JL, Rapee RM, Deveney C, Schniering CA, Lyneham HJ, Bovopoulos N. Cognitive-Behavioral Treatment Versus an Active Control for Children and Adolescents With Anxiety Disorders: A Randomized Trial. *Journal of the American Academy of Child and Adolescent Psychiatry.* 2009;48(5):533-544.

**4.** Rapee RM, Abbott MJ, Lyneham HJ. Bibliotherapy for children with anxiety disorders using written materials for parents: A randomized controlled trial. *Journal of Consulting and Clinical Psychology.* 2006;74(3):436-444.

**5.** Lyneham HJ, Rapee RM. Evaluation of therapist-supported parent-implemented CBT for anxiety disorders in rural children. *Behaviour Research and Therapy.*  2006;44(9):1287-1300.

**6.** Hudson JL, Newall C, Rapee RM, et al. The Impact of Brief Parental Anxiety Management on Child Anxiety Treatment Outcomes: A Controlled Trial. *Journal of Clinical Child and Adolescent Psychology.* 2014; 43: 370-80.

**7.** Creswell C, Singhal M, Murray L, Willetts L, Cooper P. Treatment of child anxiety in the context of maternal anxiety: a pilot study. *Clinical Psychology and Psychotherapy.* 2008;15:38–44

**8.** Lyneham HJ, Abbott MJ, Wignall A, Rapee RM. *The Cool Kids Anxiety Treatment Programme.* Sydney, Australia: Macquarie University Anxiety Research Unit; 2003.

**9.** Thirlwall K, Cooper PJ, Karalus J, Voysey M, Willetts L, Creswell C. Treatment of child anxiety disorders via guided parent-delivered cognitive-behavioural therapy: randomised controlled trial. *The British Journal of Psychiatry.* 2013;203(6):436-444.

**10.** Vassilopoulos SP, Banerjee R, Prantzalou C. Experimental modification of interpretation bias in socially anxious children: Changes in interpretation, anticipated interpersonal anxiety, and social anxiety symptoms. *Behaviour Research and Therapy.* 2009;47(12):1085-1089.

**11.** Creswell C, Hentges F, Parkinson M, Sheffield P, Willetts L, Cooper P. Feasibility of guided cognitive behaviour therapy (CBT) self-help for childhood anxiety disorders in primary care. *Mental health in family medicine.* 2010;7(1):49-57.

**12.** Rapee RM, Lyneham HJ, Schniering CA, et al. *Cool Kids "Chilled" Adolescent Anxiety Program.* Sydney, Australia: Macquarie University Anxiety Research Unit; 2006.

**13.** Barrett PM, Farrell LJ, Ollendick TH, Dadds M. Long-Term Outcomes of an Australian Universal Prevention Trial of Anxiety and Depression Symptoms in Children and Youth: An Evaluation of the Friends Program. *Journal of Clinical Child and Adolescent Psychology.* 2006;35(3):403-411.

**14.** Barrett PM. *FRIENDS for Life program - Group leader's workbook for children.* 4th ed. Brisbane, Queensland: Australian Academic Press; 2004.

**15.** Wergeland GJH, Fjermestad KW, Marin CE, et al. An effectiveness study of individual vs. group cognitive behavioral therapy for anxiety disorders in youth. *Behaviour Research and Therapy.* 2014;57(0):1-12.

**16.** Kendall PC. Treating anxiety disorders in children: Results of a randomized clinical trial. *Journal of Consulting and Clinical Psychology.* 1994;62(1):100-110.

**17.** Schneider S, Blatter-Meunier J, Herren C, et al. The efficacy of a family-based cognitive-behavioral treatment for separation anxiety disorder in children aged 8–13: A randomized comparison with a general anxiety program. *Journal of Consulting and Clinical Psychology.* 2013;81(5):932-940.

**18.** Schneider S, Lavallee K. Separation Anxiety Disorder. In: Essau CA, Ollendick TH, eds. *The Wiley-Blackwell Handbook of The Treatment of Childhood and Adolescent Anxiety*. Hoboken, NJ: Wiley-Blackwell; 2013:301-334.

**19.** Nauta MH, Scholing A. *Cognitieve gedragstherapie bij kinderen en jongeren met angststoornissen: een protocol van 12 sessies. Handleiding voor de therapeut.* Groningen: Rijksuniversiteit Groningen (Klinische en Ontwikkelingspsychologie); 1998.

**20.** Smith P, Yule W, Perrin S, Tranah T, Dalgleish TIM, Clark DM. Cognitive-Behavioral Therapy for PTSD in Children and Adolescents: A Preliminary Randomized Controlled Trial. *Journal of the American Academy of Child and Adolescent Psychiatry.* 2007;46(8):1051-1061.

**21.** Hudson JL, Lester KJ, Lewis CM, et al. Predicting outcomes following cognitive behaviour therapy in child anxiety disorders: the influence of genetic, demographic and clinical information. *Journal of Child Psychology and Psychiatry.* 2013;54(10):1086-1094.
